# Energy-Oriented Wireless Communication Platform Selection System in the Internet of Things

**DOI:** 10.3390/s26103158

**Published:** 2026-05-16

**Authors:** Konrad Gac, Jakub Gorski, Grzegorz Gora, Joanna Iwaniec

**Affiliations:** AGH University of Krakow, Faculty of Mechanical Engineering and Robotics, Department of Robotics and Mechatronics, Al. Mickiewicza 30, 30-059 Krakow, Poland; kgac@agh.edu.pl (K.G.); ggora@agh.edu.pl (G.G.); jiwaniec@agh.edu.pl (J.I.)

**Keywords:** Internet of Things, energy-efficient wireless communication, RMS current consumption, WiFi, BLE, LoRa

## Abstract

The Internet of Things (IoT) has become a fundamental paradigm in modern communication systems, enabling the large-scale interconnection of sensors, actuators, and embedded computing platforms. This paper presents a decision-oriented framework for the selection of energy-sensitive wireless communication platforms in IoT systems. The proposed approach combines systematic measurement, structured feature engineering, and lightweight regression models to predict energy consumption and current demand for different hardware platforms and wireless technologies, including ESP32- and NORA-based devices utilizing Wi-Fi, Bluetooth Low Energy (BLE) and LoRa communication. The results confirm that simple and interpretable regression models can provide robust guidance for platform and technology selection in realistic real-world scenarios, without incurring the complexity associated with detailed physical-layer or protocol-level simulations.

## 1. Introduction

The Internet of Things (IoT) has become a fundamental paradigm in modern communication systems, enabling the large-scale interconnection of sensors, actuators, and embedded computing platforms. The rapid proliferation of IoT deployments in domains such as environmental monitoring, smart infrastructure, healthcare, and industrial automation has resulted in a constantly increasing number of connected devices. A significant fraction of these devices operate under strict energy constraints, as they are powered by batteries or energy-harvesting sources and are expected to function autonomously over extended periods of time [1].

In energy-constrained IoT systems, wireless communication constitutes one of the dominant contributors to overall power consumption. Although sensing and local processing are often optimized for low-power operation, data transmission typically incurs a substantial energy overhead. Consequently, the selection and management of wireless communication technologies play a crucial role in determining device life expectancy and system sustainability. A wide range of wireless technologies are currently employed in IoT systems, including WiFi, Bluetooth Low Energy (BLE), ZigBee, NB-IoT and the Long Range Wide Area Network (LoRa/LoRaWAN), each offering different trade-offs in terms of communication range, data rate, latency, reliability, and energy consumption [1,2].

In many practical IoT deployments, the choice of communication technology is made statically at the design stage. Designers typically rely on application requirements such as nominal communication range or throughput, as well as energy consumption figures reported on manufacturers’ datasheets or application notes. Once deployed, the system runs permanently using the selected technology, without adaptation to changing channel conditions or network environments. Although this approach simplifies system design, it often leads to suboptimal energy usage in real-world scenarios where link quality and traffic patterns vary dynamically.

To better understand and optimize energy consumption, numerous studies have proposed detailed analytical and measurement-based energy models for wireless communication technologies. Time-domain and protocol-sensitive energy models have been developed for WiFi and IEEE~802.11ah [3], providing an accurate estimate of energy consumption and battery lifetime based on physical and MAC layer behavior [4,5]. Similarly, several analytical and experimental models have been introduced for LoRa and LoRaWAN, capturing the influence of transmission parameters, protocol mechanisms, and network conditions on energy performance [6,7,8]. While these approaches offer high accuracy, they typically require detailed current waveforms, protocol-level knowledge, and non-negligible computational resources, which limit their suitability for deployment on resource-constrained edge devices.

More recently, data-driven approaches have gained attention as an alternative for modeling and predicting energy consumption in IoT networks. Regression and machine-learning-based models have been applied to forecast energy consumption and reliability metrics using communication parameters such as packet size, transmission power, and link quality indicators [9,10]. Although advanced models, including deep learning techniques, can achieve high prediction accuracy, their computational and memory requirements often exceed the capabilities of low-power embedded platforms. Consequently, recent studies have increasingly focused on lightweight and energy-aware machine-learning solutions suitable for resource-constrained IoT edge devices, including TinyML and low-complexity adaptive decision systems [11,12].

In parallel to energy modeling efforts, adaptive communication and radio access technology selection mechanisms have been investigated, primarily in heterogeneous wireless networks. Existing solutions focus mainly on performance optimization, such as throughput, latency, or reliability, using centralized optimization, multi-criteria decision-making, or learning-based approaches [13,14]. Energy-aware selection mechanisms remain comparatively underexplored, particularly in the context of lightweight implementations suitable for edge devices of the IoT.

In this work, an energy-oriented wireless communication platform selection system is designed for edge nodes of the IoT. The proposed system relies on lightweight linear regression models to predict energy consumption and current demand for different hardware platforms and wireless technologies, including ESP32- and NORA-based devices utilizing Wi-Fi, Bluetooth Low Energy (BLE) and LoRa communication. Unlike detailed physical-layer energy models, the proposed approach is based on aggregated RMS current measurements rather than high-resolution current waveforms. This design choice significantly reduces the complexity and computational overhead of the model, enabling practical implementation in resource-constrained edge devices.

The proposed system represents a compromise between static, datasheet-based communication selection and highly accurate but computationally expensive analytical energy models. By enabling adaptive selection of the communication medium based on predicted energy consumption under current link conditions, the proposed approach introduces runtime flexibility while preserving feasibility for embedded IoT platforms. The main contribution of this article is the design, implementation, and evaluation of this regression-based communication platform selection framework, demonstrating its potential to improve energy efficiency in multi-radio IoT systems. A comparison of representative related works and their main limitations with respect to the proposed approach is presented in Table 1.

The remainder of this paper is organized as follows. Section 2 presents the experimental multi-radio IoT system supporting BLE, Wi-Fi, and LoRa communication. Section 3 describes the measurement procedure and dataset design, while Section 4 provides an exploratory analysis of the collected data. Section 5 introduces the proposed communication platform selection framework. Section 6 and Section 7 describe the feature engineering process, regression models, and training procedures. Section 8 evaluates the developed current consumption regressors, and Section 9 introduces the proposed platform selection evaluation metrics. Section 10 presents the platform selection results and ablation analysis, while Section 11 discusses practical use cases of the proposed system. Section 12 addresses the limitations and discusses the results obtained. Finally, Section 13 concludes the paper and summarizes the main findings.

## 2. Materials and Methods

**Figure 1:**
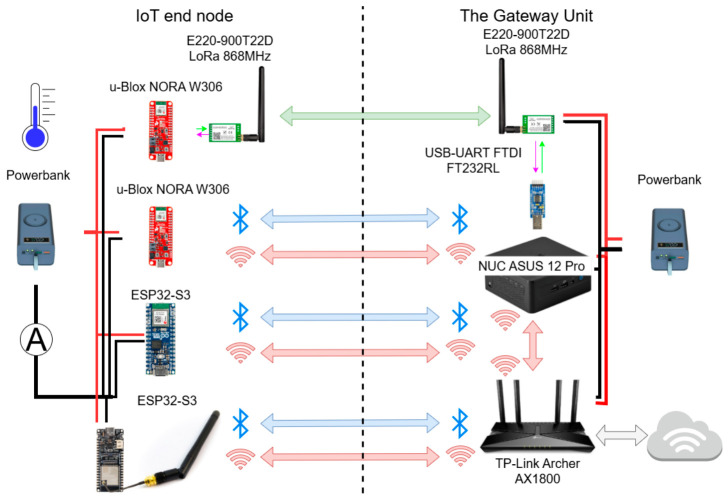
Diagram of the built IoT system for measuring energy demand.

The measurement setup used in the experiments conducted consisted of two independent subsystems: the IoT Gateway Unit and the IoT end node. A block diagram of the measurement setup developed is presented in Figure 1.

### 2.1. System Architecture for Wireless Communication

The Gateway Unit was built using the ASUS NUC 12 Pro platform (ASUSTeK Computer Inc., Taipei, Taiwan), which served as the central processing and coordination unit of the measurement system. Wi-Fi communication within the system was provided through a network managed by a TP-Link Archer AX1800 router (TP-Link Technologies Co., Ltd., Shenzhen, China). For Bluetooth Low Energy (BLE) communication, the ASUS platform operated as the central node, enabling direct interaction with the BLE-based IoT devices. In addition, a LoRa E220-900T22D (Ebyte, Chengdu, China) module was connected to the ASUS computer via a USB-to-UART converter, allowing integration with long-range communication links. The gateway device was powered by a portable power bank, enabling full mobile operation and facilitating measurement campaigns conducted in outdoor and field environments.

The modules comprising the IoT end node were composed of two embedded hardware platforms. The first platform was based on the chip ESP32-S3 system (DFRobot, Shanghai, China) and was evaluated in two antenna configurations: an integrated printed circuit board (PCB) antenna and an external antenna (Ext). In both configurations, the ESP32 platform supported WiFi and Bluetooth Low Energy (BLE) communication, enabling a consistent assessment of short- and medium-range wireless technologies under identical processing conditions.

The second platform was based on the NORA W306 module (SparkFun Electronics, Niwot, CO, USA; u-blox, Thalwil, Switzerland) equipped with an integrated PCB antenna and natively supporting WiFi and BLE. To extend the experimental scope to wide-area communication, LoRa connectivity was provided through an external E220-900T22D radio module interfaced with the u-Blox platform. This configuration enabled the evaluation of WiFi, BLE, and LoRa within a unified experimental framework while maintaining a clear separation between platform-level processing and radio-specific functionality. The end node was also powered by a power bank to ensure autonomous operation independent of external power sources. Furthermore, to enable analysis of energy performance during the measurements, the sensor unit was fitted with a current consumption monitoring module.

### 2.2. Wireless Communication Technologies

In this study, three representative wireless communication technologies were selected to reflect different design trade-offs commonly encountered in Internet of Things (IoT) systems: Wi-Fi, BLE, and LoRa. These technologies span a broad spectrum in terms of throughput, communication range, protocol complexity, and energy consumption characteristics.

Wi-Fi, which belongs to the IEEE 802.11 family of standards, was included as a high-throughput wireless technology widely adopted in data-intensive IoT applications. Conventional Wi-Fi variants such as IEEE 802.11g and IEEE 802.11n operate primarily in the 2.4 GHz and 5 GHz industrial, scientific and medical (ISM) bands and offer data rates ranging from several up to hundreds of megabits per second, depending on the modulation employed and channel bandwidth [15]. More recent standards, including IEEE~802.11ax, further increase spectral efficiency and achievable data rates at the cost of increased protocol complexity. Although Wi-Fi provides high throughput and low latency, it is characterized by relatively high energy consumption, primarily due to connection establishment procedures, medium contention mechanisms, and protocol overhead, which can significantly impact battery-powered IoT devices [4,5].

Bluetooth Low Energy (BLE) represents a short-range wireless technology specifically optimized for low-power operation and sporadic data transmission. Standardized as part of Bluetooth 4.0 and further enhanced in Bluetooth 5.x, BLE operates in the 2.4 GHz ISM band using narrowband channels and lightweight protocol mechanisms [16]. Typical indoor communication ranges from 10 to 100 m, depending on transmission power and environmental conditions. Bluetooth 5 introduced optional coded physical layer modes that extend the communication range to several hundred meters in open-space scenarios while maintaining low energy consumption [17]. Due to its low current consumption during transmission and idle states, BLE is particularly well suited for energy-constrained IoT nodes with moderate throughput requirements, such as wearable devices and local monitoring systems.

LoRa (Long Range) was selected as a representative low-power wide-area network (LPWAN) technology designed for long-range communication with minimal energy consumption. Operating in unlicensed sub-GHz ISM bands, such as 868 MHz in Europe and 915 MHz in North America, LoRa employs chirp spread spectrum modulation to achieve high link robustness and extended communication range [18]. Depending on environmental conditions, the LoRa links can reach several kilometers in urban areas and more than 15 km in rural or open-space environments. However, this extension of the range comes at the cost of very low data rates, typically between 0.3 and 50 kbps, and prolonged transmission times, which influence overall energy consumption despite low instantaneous current draw [7,8].

The comparison was conducted at the system level, without analyzing the physical-layer or modulation-specific details. This design choice reflects the intended application of the proposed model, which aims to support adaptive technology selection based on aggregated operational metrics rather than detailed radio internals.

## 3. Measurement Procedure and Dataset Design

The dataset was developed through a series of controlled measurement experiments designed to capture the relationship between transmission parameters, radio propagation conditions, and the energy consumption of the analyzed communication platforms.

### 3.1. Experiment Description

**Figure 2:**
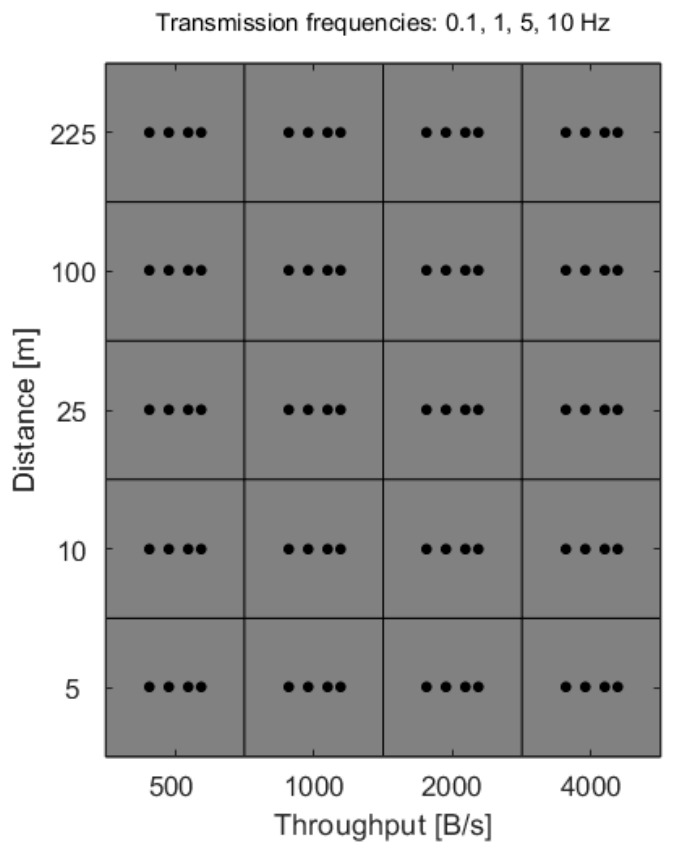
Diagram of the Cartesian product of parameters.

Experiments were conducted for a total of 80 different operational configurations obtained as combinations of three primary control variables. The distance between the IoT node and the network gateway was set to discrete values of 5, 10, 25, 100, and 225 m. Transmission throughput was configured at 500, 1000, 2000, and 4000 B/s, while the transmission frequency, defined as the rate at which data samples were sent, took values of 0.1, 1, 5 and 10 Hz. During measurements using the Wi-Fi network, the TCP/IP protocol was used. The complete Cartesian product of these parameters resulted in 80 unique operating points presented on the grid diagram in Figure 2.

For each configuration, measurements were performed for all the communication platforms analyzed. The current consumption of the RMS was measured over fixed transmission intervals corresponding to steady-state operation, so each measurement sample represented a stable operating point rather than a transient condition. Instantaneous current waveform analysis was not performed; instead, the use of RMS current values was a deliberate methodological choice that reflected the energy consumption at the system level as perceived by the power supply and battery subsystem.

During each experiment, instantaneous RSSI values were recorded independently for each communication platform. These raw RSSI measurements were subsequently aggregated to derive statistical descriptors characterizing link quality for a given configuration, specifically the median RSSI and its standard deviation. The median value was selected to reduce the sensitivity to outliers, while the standard deviation was included to capture link variability over time, which is known to influence retransmissions and protocol behavior.

In addition to radio metrics, the ambient temperature at the IoT node was measured throughout the experiments and treated as an environmental parameter that influences the electrical characteristics and energy consumption of the system. Although the temperature range was limited by the duration of the measurement campaign to two days, no alternative parameter was identified that could adequately substitute for its effect; therefore, the temperature was retained as a model input variable.

### 3.2. Dataset Construction

In total, 33,709 raw data samples were collected during the measurement campaign. The measured parameters together with their corresponding ranges are summarized in Table 2. The selected parameter ranges were designed to reflect realistic operating conditions encountered in practical IoT deployments, including short- and medium-range communication scenarios, varying radio propagation conditions, and different traffic intensities. To ensure the validity of the collected dataset, successful transmission of 100% of the data frames was verified for each measurement case.

These data were subjected to preprocessing steps that included aggregation and feature extraction, resulting in a compact dataset that comprises 80 data points, each corresponding to a unique operational configuration. For each data point, the extracted features described the statistical properties of the radio link, traffic characteristics, and environmental conditions, while the target variable was defined as the RMS current consumption associated with that configuration.

## 4. Exploratory Data Analysis

In order to preliminarily assess the relationship between the experimental parameters and the current demand, the measurement data was subjected to exploratory analysis based on graphical visualization. The aim of this stage was to identify the dominant trends, potential non-linearities, and factors that have the greatest impact on the energy consumption of individual communication platforms.

### 4.1. Distribution of Current Consumption

**Figure 3:**
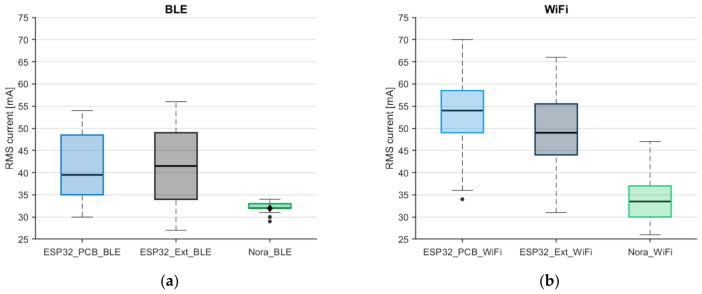
Comparison of current consumption for different platforms when using: (**a**) BLE, (**b**) WiFi. The circles are outliers and the diamond are median.

The first step contained a comparative assessment of current consumption across the analyzed communication platforms. For this purpose, Figure 3 presents box plots for the ESP32 platform with an integrated PCB and with an external antenna and the NORA platform with an integrated antenna, considered for two communication technologies: BLE and WiFi.

The analysis reveals a clear and consistent difference between the analyzed platforms. For both communication technologies, the NORA platform exhibits lower median current consumption values, indicating a more energy-efficient operating profile. These differences are evident not only in the central values of the distributions but also in the upper quartiles, which are particularly relevant in scenarios characterized by increased energy demand.

When comparing the two variants of ESP32 (PCB antenna versus external antenna), the observed differences in current consumption are relatively small. The corresponding distributions overlap a lot, and differences in medians and quartiles do not indicate a clear energy advantage of one variant over the other.

### 4.2. Influence of Communication Parameters on Current Consumption

**Figure 4:**
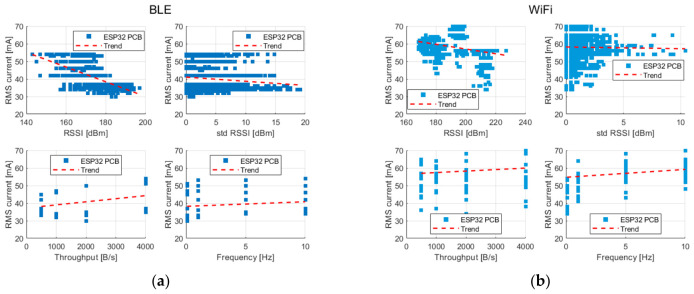

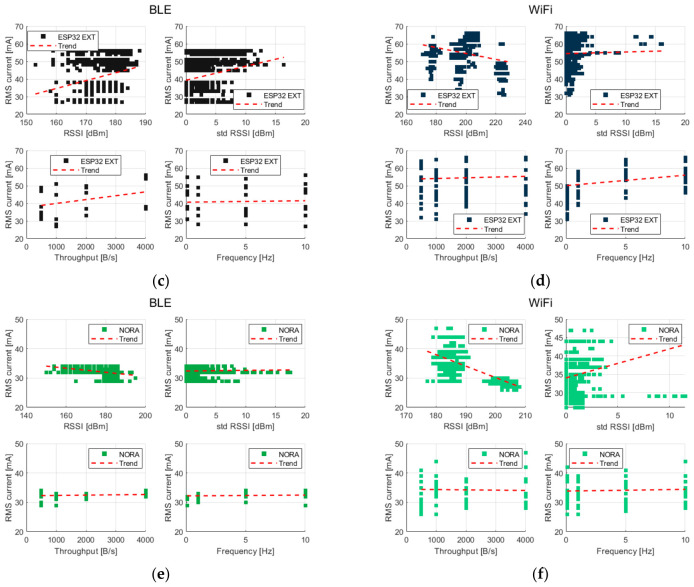
Scatter plots showing the relationships between measured transmission parameters and the RMS current value for the following platforms: (**a**) ESP32 PCB BLE, (**b**) ESP32 PCB WiFi, (**c**) ESP32 Ext BLE, (**d**) ESP32 Ext WiFi, (**e**) NORA BLE, (**f**) NORA WiFi.

Further analyses were conducted to observe the impact of the measured parameters on energy demand. Figure 4 illustrates the relationships between current consumption and selected transmission and link-quality parameters, including RSSI, the standard deviation of RSSI, transmission throughput, and transmission frequency. The analysis was conducted separately for each hardware platform and for both BLE and WiFi technologies to account for the baseline energy characteristics of the platform and protocol-dependent behavior.

For WiFi-based communication projected in Figure 4b,d,f, a clear influence of RSSI on the current consumption can be observed. As RSSI increases, the RMS current generally decreases, which is physically justified by improved link quality, resulting in fewer retransmissions and reduced protocol overhead. However, the observed relationship is not strictly linear. Instead, it exhibits a monotonic but distinctly non-linear trend, indicating that improvements in signal quality yield diminishing energy benefits at higher RSSI values.

In contrast, for the BLE-based communication presented in Figure 4a,c,e, the effect of RSSI on current consumption is less pronounced. The corresponding plots do not reveal a clear increase or decrease trend, and variations in RSSI appear to have less influence on the RMS current.

Transmission throughput has a more significant role in the case of BLE. An increase in throughput is associated with a higher current consumption, indicating that the offered traffic load directly affects the energy consumption of BLE-based platforms. For WiFi, throughput does not exhibit a similarly strong influence on current consumption. Instead, the transmission frequency emerges as a more relevant factor. An increase in transmission frequency leads to a noticeable rise in RMS current, which can be attributed to repeated connection establishment and teardown cycles after wake-up and data transmission.

The influence of the standard deviation of RSSI on current consumption is less straightforward and varies between platforms and technologies. No uniform trend can be identified in all cases. However, among the configurations analyzed, the steepest trend is observed with respect to RSSI variability for the NORA platform that operates with WiFi. This may suggest that link-quality fluctuations may have a more pronounced impact on the energy behavior of this platform, potentially due to differences in radio implementation or protocol handling.

**Figure 5:**
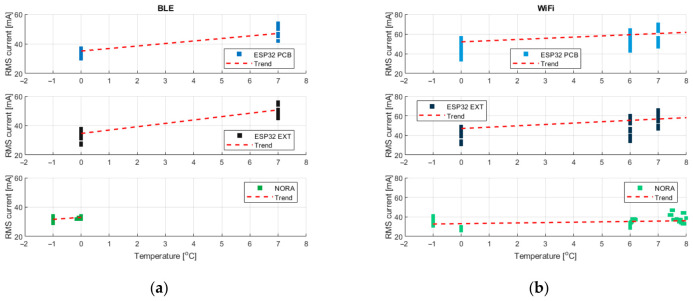
Scatter plots showing the relationships between measured ambient temperature and the RMS current value for the following media: (**a**) BLE, (**b**) WiFi.

Figure 5, illustrating the relationship between ambient temperature and current consumption, reveals a different pattern. An increasing trend in current consumption with increasing temperature is visible, particularly for ESP32-based platforms. This suggests that temperature has a noticeable effect on the energy characteristics of these devices. For the NORA platform, a similar trend can be observed, although its magnitude appears significantly smaller, indicating a reduced sensitivity to temperature variations.

**Figure 6:**
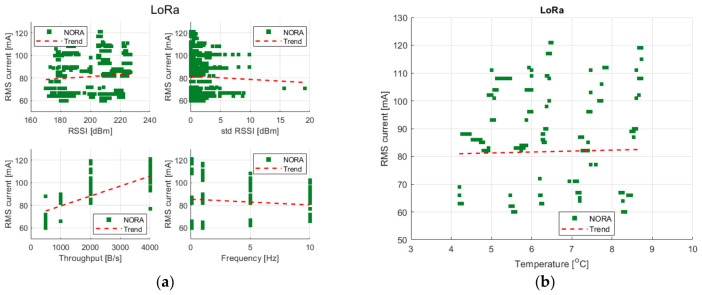
Scatter plots showing the relationships between measured transmission parameters: (**a**) RSSI, std RSSI, bandwidth, and frequency; (**b**) ambient temperature vs. the RMS current value for LoRa.

The graphs corresponding to the LoRa technology presented in Figure 6a illustrate the relationships between the current consumption and selected transmission parameters, including RSSI, the standard deviation of RSSI, transmission throughput and transmission frequency.

Neither RSSI nor the standard deviation of RSSI exhibits a clear or consistent trend with respect to the current consumption. The corresponding scatter plots remain largely flat, indicating that variations in received signal strength and its temporal variability do not directly translate into measurable changes in current consumption within the operating range analyzed.

In contrast, transmission throughput and transmission frequency play a dominant role in shaping the energy consumption of LoRa-based systems. An increase in throughput is associated with a higher current consumption, reflecting the higher amount of data transmitted per unit of time. These observations are consistent with the underlying principles of the physical layer.

The final parameter considered is the ambient temperature, as shown in Figure 6b. For LoRa-based platforms, no clear relationship between temperature and current consumption can be identified within the analyzed temperature range. The observed trends remain nearly flat, indicating that the effects related to temperature are marginal compared to other dominant factors that govern energy consumption in this scenario.

## 5. System Overview

**Figure 7:**
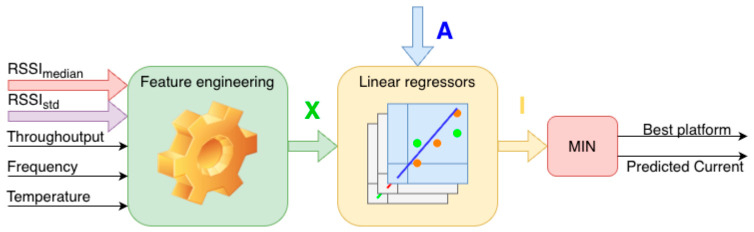
Block diagram showing the structure of the platform selection system.

The proposed system performs the task of estimating the current consumption for the available wireless communication platforms and selecting the optimal energetic platform under given environmental and transmission conditions. The system architecture is composed of several interconnected processing blocks, whose interaction is illustrated in Figure 7.

The input to the system consists of a set of features that describe radio propagation conditions, transmission configuration, and environmental state. These features include the median RSSI value measured for each transmission platform, the standard deviation of RSSI capturing temporal variability of the radio link, the transmission throughput, the transmission frequency defined as the rate of sample transmission, and the ambient temperature measured at the IoT node. All base features are registered independently for each analyzed platform and form the initial representation of the operating conditions.

All primary features are preserved and used as input to the feature engineering stage. At this stage, an extended feature vector X is constructed through a systematic process of feature transformation, combination, and aggregation applied to the base variables. The complete composition of the feature vector X and the rationale behind the selected transformations are described in detail in Section 6.

The extended feature vector X serves as the direct input to a set of linear regression models. Each regressor is based on a linear regression formulation and is dedicated to a single, specific communication platform. Regressors are activated by a binary vector A, which provides information about the availability of a given platform. A detailed description of the regressor architecture, training procedure, and standardization process is provided in Section 7.

The output of this stage is a vector of predicted current values I corresponding to the candidate platforms. In the final decision step, a minimum-selection operation is applied to identify the platform characterized by the lowest predicted current consumption. The main actions of the described procedure are presented using Algorithm 1.

As a result, the system produces two outputs: the identifier of the communication platform that is expected to be energetically optimal for the given operating conditions and the predicted current consumption associated with this platform.
**Algorithm 1** Proposed platform selection system
**Input:** RSSI samples **R**, Throughput **T**, Frequency **F**, Temperature **Temp**
**Output:** Best platform **p^sel^**, Predicted current **I_p_****1**Update sliding RSSI window **W_RSSI_** with the newest RSSI sample **R****2**Remove the oldest RSSI sample from **W_RSSI_****3**Compute **RSSI_median_** = median(**W_RSSI_**)**4**Compute **RSSI_std_** = std(**W_RSSI_**)**5**Construct feature vector **X** using **RSSI_median_**, **RSSI_std_**, **T**, **F**, and **Temp****6**Apply feature engineering to generate derived features**7****For each** platform **p_i_ do****8****|**   Predict current consumption **I_pi_ = f_pi_(X)****9****End for****10**Select platform **p^sel^ =arg min(I)**

## 6. Feature Engineering

The feature engineering stage is responsible for expanding the input feature space by generating additional variables computed from the measured quantities.

The base set consists of directly measured or aggregated quantities describing radio link conditions, traffic characteristics, and the environmental context presented in Table 3.

These include the median RSSI value, the standard deviation of RSSI, the transmission throughput expressed in B/s, the transmission frequency defined as the rate of transmission of the sample in Hz, and the ambient temperature measured at the IoT node. The median RSSI was selected to provide a robust estimate of signal strength that is less sensitive to outliers, while the standard deviation of RSSI captures the temporal variability of the radio link, which is known to influence the retransmission behavior and the protocol overhead. Throughput and transmission frequency jointly characterize the offered traffic load, while temperature accounts for environmental effects on electronic components and radio performance.

To enable a linear model to approximate non-linear relationships inherent in wireless energy consumption, a set of derived and interaction features was introduced. The complete set of derived features is summarized in Table 4.

The squared RSSI term was introduced to reflect the non-linear relationship between signal quality and energy demand, where deteriorating propagation conditions can lead to a disproportionate increase in energy consumption due to longer transmission times, retransmissions, or adaptive protocol behavior [19].

Logarithmic transformations of throughput and transmission frequency were applied to reduce skewness in feature distributions and to better reflect the diminishing marginal impact of increasing traffic intensity on energy consumption [20].

The ratio of transmission frequency to throughput provides a normalized measure of transmission load, which captures how frequently communication events occur relative to the amount of transmitted data [21].

The product of median RSSI and throughput describes the interplay between channel quality and data volume, while the interaction between temperature and transmission frequency allows the model to account for the joint influence of environmental conditions and operational intensity [22].

All derived features, together with the base features, form an extended input vector X that serves as the direct input to the linear regression models. The candidate feature set was intentionally restricted to variables that are both measurable during operation and actionable from a system design or configuration perspective.

Prior to model training, the dataset was subjected to a preprocessing stage. Missing values occurred primarily in scenarios where a given communication technology became unavailable due to excessive distance between the IoT node and the gateway. Since such cases do not represent valid operating conditions, the corresponding observations were removed from the dataset rather than imputed.

To reduce the influence of outliers and short-term fluctuations in radio measurements, raw values such as RSSI were not used directly. Instead, a filtering approach was applied to obtain robust estimates of RSSI_median_ or RSSI_std_ using a sliding window of five consecutive samples.

## 7. Regression Models and Training Procedure

Regression models constitute the core component of the proposed system and are responsible for the quantitative estimation of current consumption for the available communication platforms. Linear regression was selected as the base modeling approach due to its favorable balance between simplicity and practical relevance.

Although the feature engineering stage produces an extended input space consisting of both base and derived variables, not all features are assumed to be equally relevant for every hardware platform and communication technology. Therefore, to improve robustness and promote sparsity in learned parameters, the regularization of LASSO was used based on the norm L1, allowing automatic selection of features while reducing the risk of overfitting [23]. The L1 penalty promotes sparse solutions by shrinking the coefficients of less informative or redundant features to zero due to the use of the absolute-value regularization term.

In the study considered, seven communication platforms were analyzed, each associated with an independent regression model trained exclusively on data corresponding to a specific hardware configuration and communication technology. The set of platforms is defined as

(1) p∈{ESP32 PCB BLE; ESP32 PCB WiFi; ESP32 Ext BLE;ESP32 Ext WiFi; Nora BLE; Nora WiFi; Nora LoRa},

The direct input to each regression model is the extended characteristic vector X obtained after the feature engineering stage.

Before regression, all features are standardized using z-score normalization according to

(2) $$X_{n}=\frac{X-\mu }{\sigma }$$

where μ denotes the vector of mean values of individual features and σ denotes the corresponding vector of standard deviations. The normalization parameters μ and σ are computed exclusively in the training subset and stored as fixed parameters for each regressor.

For a given platform pi, the predicted current consumption I_pi_ is calculated as a linear function of the normalized feature vector,

(3) $$I_{pi}=\beta_{pi}^{T}X_{n}+\beta_{0pi}$$

where β_pi_ is the vector of regression coefficients learned during training and β_0pi_ is the bias term. The parameter vector β_pi_ is estimated by minimizing a LASSO-regularized least-squares objective,

(4) $$\beta_{pi}=\arg\underset{\beta_{\mathrm{pi}},\beta_{0\mathrm{pi}}}{\min}\left({\left\|Y_{pi}-\beta_{0pi}-X_{n}\beta_{pi}\right\|}^{2}+\lambda_{i}\left\|\beta_{pi}\right\|\right)$$

where Y_pi_ denotes the vector of measured RMS current values for platform pi and λ_i_ is the regularization coefficient controlling sparsity. The regularization parameter λ_i_ was determined via k-fold cross-validation, selecting the value that minimized the validation error while favoring sparse models suitable for deployment on resource-constrained edge devices.

Prediction is performed only on platforms that are deemed available at a given processing stage. Platform availability is encoded in a binary availability vector,

(5) $$A=\left[A_{p1},A_{p2},\ldots A_{p7}\right],A_{pi}\in \left\{0,1\right\},i=1,2,\ldots ,7$$

where A_pi_ = 1 indicates that the platform p is available and eligible for prediction. For platforms with A_pi_ = 0, the regression model is not evaluated, and the platform is excluded from further consideration. For available platforms, the regression block produces a vector of predicted current values,

(6) $$I=\left[I_{p1},I_{p2},\ldots I_{p7}\right]$$

which is subsequently used in the decision-making stage to select the energetically optimal communication platform.

**Figure 8:**
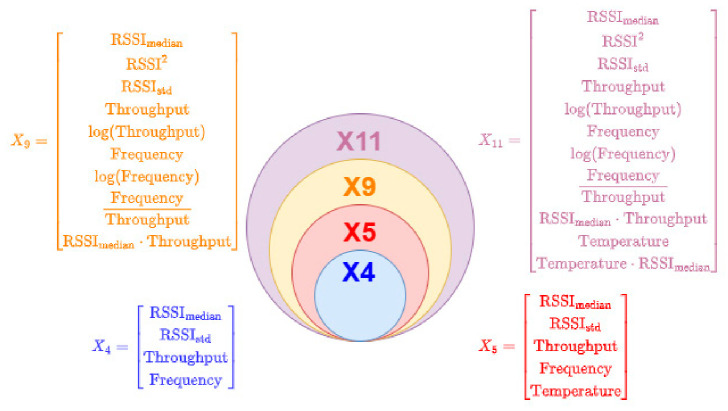
Feature set composition (X4, X5, X9, X11).

To assess the impact of feature selection and model complexity, four distinct feature sets were defined and evaluated as presented in Figure 8. The X4 feature set includes only linear base features excluding temperature. The X5 feature set extends X4 by including temperature as an additional linear feature. The feature set X9 incorporates non-linear and interaction features while excluding temperature and its derived terms. Finally, the X11 feature set comprises the complete set of base, non-linear, and interaction features. This structured ablation design enables a systematic evaluation of the contribution of individual feature groups to predictive performance.

Model training and validation followed a consistent procedure across all platforms and feature sets. For each platform, the dataset was randomly partitioned into a training subset containing 80% of the data and a test subset containing the remaining 20%. All parameters appearing in (2) to (4) were estimated exclusively in the training data.

## 8. Evaluation of Current Regressors

The aggregated evaluation results are presented in the form of box plots, computed separately for each current regressor corresponding to the analyzed platforms. For each regressor, the RMSE value was obtained through a repeated evaluation procedure consisting of 50 independent iterations, each involving a random split of the dataset into training and test subsets with a ratio of 80% to 20%, followed by model training and performance evaluation.

**Figure 9:**
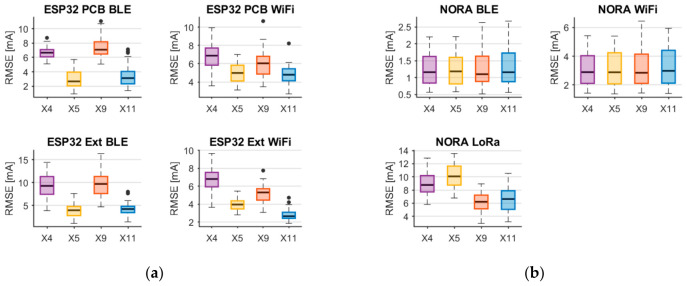
RMSE values for the evaluation of regression models developed for the following platforms: (**a**) ESP32 PCB and Ext; (**b**) NORA. The circles are outliers.

The results are summarized in two graphs: Figure 9a shows the distributions of the RMSE values for the ESP32-based platforms, while Figure 9b presents the corresponding results for the NORA platform. Each box plot compares the performance of models trained with different feature sets, namely X4, X5, X9, and X11.

For the ESP32 platform, the results clearly confirm the importance of temperature as an explanatory variable. Incorporating temperature into the feature set, as in X5, leads to a statistically meaningful reduction in RMSE compared to both the baseline linear feature set X4, which excludes temperature, and the non-linear feature set X9, which also omits temperature-related information. This effect is particularly pronounced for ESP32 operating with WiFi, where the lowest RMSE values are achieved by models using the full feature set X11, combining non-linear features with temperature-dependent terms.

In the case of ESP32 operating with BLE, the results are less consistent. Although temperature-aware models still tend to outperform those without temperature information, the lowest median RMSE values are observed for regressors using the X5 feature set. This suggests that, for BLE implemented on ESP32, the inclusion of temperature is beneficial, whereas additional non-linear transformations provide limited further improvement under the analyzed conditions.

For the NORA platform, a different pattern emerges. For both BLE and WiFi, no consistent reduction in prediction error can be observed when temperature and non-linear factors are included. This indicates that within the dataset considered, they have a relatively minor influence on the current consumption of the NORA platform.

In contrast, a clear impact of non-linear features is observed for the LoRa-based configuration of the NORA platform. Models relying solely on linear features are unable to adequately capture the relationship between transmission parameters and current consumption, resulting in higher RMSE values. The inclusion of non-linear feature sets, as in X9 and X11, leads to a noticeable reduction in prediction error, demonstrating that non-linear modeling components are essential for accurately representing the energy behavior of LoRa communication.

To complement the linear regression analysis, a non-linear model based on Random Forest was introduced. This choice was motivated by its ability to capture non-linear relationships while remaining suitable for deployment on resource-constrained edge devices.

Due to the limited dataset size, the model complexity was constrained to reduce overfitting. The models were trained using the X11 feature set, and three ensemble sizes (5, 10, and 20 trees) were evaluated with a maximum tree depth of 5. Separate models were trained for each platform, following the same evaluation procedure as for linear regression.

**Figure 10:**
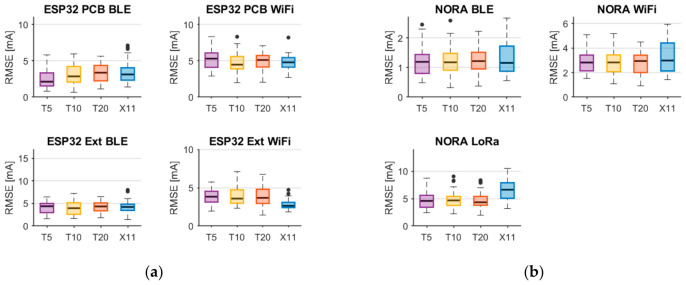
RMSE values for the evaluation of random forest models with different tree numbers developed for the following platforms: (**a**) ESP32 PCB and Ext; (**b**) NORA. The circles are outliers.

The results summarized in Figure 10a show the distributions of the RMSE values for the ESP32-based platforms, while Figure 9b presents the corresponding results for the NORA platform. The presented box plots indicate that the lowest RMSE values were typically obtained for modeling with 10 trees (T10). Increasing the number of trees did not result in statistically significant improvements, suggesting early performance saturation. Therefore, the 10-tree configuration was selected for further evaluation.

A comparison with linear regression models using the X11 feature set shows that both approaches achieve similar prediction accuracy on the test sets. This indicates that the considered relationships can be effectively approximated using linear models with engineered features.

## 9. Platform Selection Evaluation Metrics

The performance of the proposed whole selection system was evaluated using a set of metrics designed to assess not only the precision of the prediction but also primarily the quality of the energy-related decisions enabled by the regression models.

Let i = 1…N denote the index of test samples. Let $I_{i}^{ref}$ represent the measured current consumption of the energy-optimal (reference) platform for the sample i, defined as the platform with minimal measured current. Let $I_{i}^{sel}$ denote the measured current consumption of the platform selected by the system based on the predictions of the model. Furthermore, let ${\hat{I}}_{i,p}\;$ denote the predicted current consumption for platform p and sample i. The platform selected by the system is given by

(7) $$p_{i}^{sel}=\arg\underset{p}{\min}{\hat{I}}_{i,p}$$

while the reference platform derived from measurements is defined as

(8) $$p_{i}^{ref}=\arg\underset{p}{\min}I_{i,p}$$

The first evaluation metric, Cost Accuracy, quantifies the fraction of test samples for which the system selected the energetically optimal platform. It is defined as

(9) $$Cost\;Accuracy=\frac{1}{N}\sum_{i=1}^{N}1(p_{i}^{sel}=p_{i}^{ref})$$

where 1(.) denotes the indicator function. This metric provides a direct measure of the correctness of the decision from an energy-optimality perspective, independent of the magnitude of the prediction errors.

While Cost Accuracy captures whether the correct platform is chosen, it does not quantify the severity of incorrect decisions. To address this limitation, the Mean Relative Cost is introduced to measure the average percentage increase in energy consumption resulting from suboptimal platform selection. It is defined as

(10) $$Mean\;Relative\;Cost\;[\%]=\frac{100}{N}\sum_{i=1}^{N}\frac{I_{i}^{sel}-I_{i}^{ref}}{I_{i}^{ref}}$$

This metric reflects the expected energy penalty associated with system decisions and is directly interpretable in terms of relative energy overhead.

To further characterize the tail behavior of the decision-induced energy loss, the 95th percentile of the relative cost distribution is evaluated. The P95 Relative Cost captures rare but potentially critical cases in which the system selects a significantly suboptimal platform. It is defined as

(11) $$P95\;Relative\;Cost\;[\%]=P95\left(100\cdot \frac{I_{i}^{sel}-I_{i}^{ref}}{I_{i}^{ref}}\right)$$

where P95(.) denotes the 95th percentile operator. This metric is particularly relevant for applications where the worst-case energy inefficiencies must be bounded.

Finally, the Energy Loss Ratio provides an aggregate measure of the total additional energy consumption incurred by the system relative to the theoretical minimum achievable energy consumption. It is defined as

(12) $$Energy\;Loss\;Ratio=\frac{\sum_{i=1}^{N}I_{i}^{sel}-I_{i}^{ref}}{\sum_{i=1}^{N}I_{i}^{ref}}$$

This metric reflects the cumulative energy inefficiency of the system throughout the test set and is particularly suitable for evaluating long-term operational impact, such as reducing battery lifetime.

The use of the metrics defined in (9) to (12) is motivated by the decision-making nature of the problem under consideration. In contrast to classical regression error measures, these metrics explicitly account for the fact that not all prediction errors lead to equivalent energy consequences. As a result, they provide a more meaningful assessment of system performance from an engineering perspective.

## 10. Platform Selection Results and Ablation Analysis

**Figure 11:**
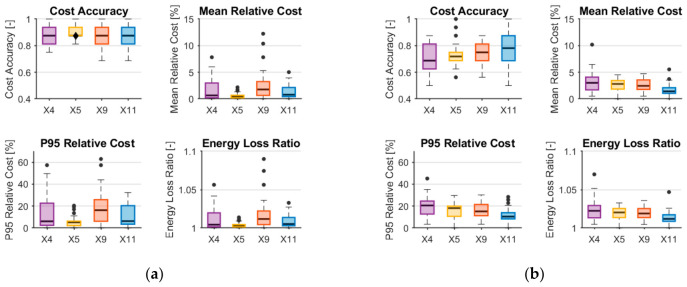
Metrics for evaluating platform selection system with linear regressor when (**a**) all platforms are included; (**b**) only ESP32 and LoRa are included. The circles are outliers.

The results of the experimental evaluation and feature ablation analysis are presented in Figure 11a,b. To ensure statistical reliability of the conclusions, the evaluation procedure was repeated 50 times, each time using a random split of the dataset into training and test subsets with a ratio of 80% to 20%. For each iteration, the regression models were trained in the training subset and subsequently used to estimate the current consumption of all the communication platforms available in the test subset.

Based on the predicted current consumption, the platform selection mechanism identified the platform associated with the minimum predicted current. The selected platform and its corresponding predicted current value were then evaluated against the reference measurements, and the outcome of the decision was assessed using the performance metrics defined in Section 9.

The results obtained considering all available communication platforms are shown in Figure 11a. Across all evaluated feature sets, from X4 to X11, the observed Cost Accuracy remains at a comparable level and exhibits only limited variability, indicating stable system behavior regardless of the specific feature configuration. Similarly, the distributions of the mean relative cost, P95, and the energy loss ratio show a relatively small dispersion in repeated runs.

In particular, the overall performance of the platform selection remains comparable between all feature sets in this scenario. This observation can be explained by the structure of the experimental dataset. During the measurement campaign, the lowest energy consumption was consistently observed for NORA family-based platforms, particularly for configurations where WiFi and BLE were used. For these platforms, no strong dependency was observed between energy consumption and the transmission parameters analyzed. As a result, even relatively simple models operating on a reduced feature set, such as X4, are sufficient to correctly identify the energetically optimal platform in most cases. Consequently, the inclusion of additional non-linear or interaction features does not significantly improve Cost Accuracy when all platforms are included, as the decision problem is dominated by a subset of platforms with consistently minimal energy demand.

To provide a more detailed view of the impact of feature engineering and model complexity, an additional evaluation scenario was considered. In this variant, NORA platforms that operate in WiFi and BLE were deliberately excluded from the candidate set. This modification removes the dominant low-energy platforms and exposes a more energetically diverse group of alternatives. The results of this evaluation are shown in Figure 11b.

Under these conditions, the influence of the composition of the set of characteristics becomes significantly more pronounced. Models based on the simplified X4 feature set exhibit noticeably lower Cost Accuracy and higher values of Mean Relative Cost, P95 Relative Cost, and Energy Loss Ratio. As progressively richer feature sets are introduced, from X5 through X9 to X11, a systematic improvement in performance is observed. In particular, the inclusion of non-linear and interaction features leads to a more accurate ranking of candidate platforms and a consistent reduction in the energy penalty associated with suboptimal decisions. The second evaluation scenario particularly highlights the importance of model complexity under conditions where the energy differences between candidate communication platforms become less pronounced.

The improvement is especially evident in tail-oriented metrics such as P95 Relative Cost, where richer feature representations substantially reduce the magnitude of worst-case energy losses. This indicates that extended feature sets not only improve average decision quality but also enhance robustness against unfavorable operating conditions.

From a technology-specific perspective, the results highlight distinct behavior across the WiFi, BLE, and LoRa platforms. When the platform energy characteristics are clearly separated, as in the case of the full set of platforms, simple features are sufficient for the correct selection. However, when energy differences are more subtle and depend strongly on operating conditions, as in the reduced set of platforms, richer feature representations become critical.

**Figure 12:**
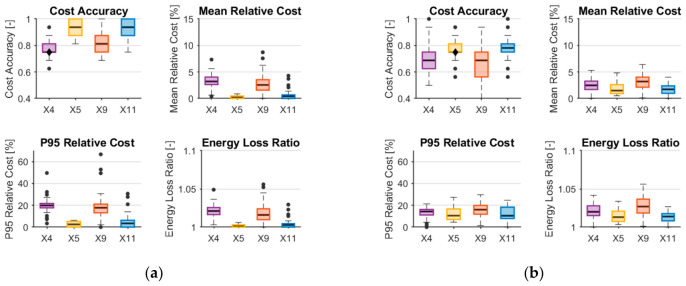
Metrics for evaluating platform selection system with random forest regressor when (**a**) all platforms are included; (**b**) only ESP32 and LoRa are included. The circles are outliers.

To further extend the analysis, the platform selection system was evaluated using a non-linear model based on Random Forest. The models were configured with 10 trees and a maximum depth of 5 to balance prediction performance and edge deployment constraints. Separate regressors were trained for each platform using feature sets from X4 to X11, and the evaluation followed the same procedure and metrics as in the linear case. The results are presented in Figure 12.

For the full set of platforms, Random Forest models show improved decision quality when temperature-related features are included (X5 and X11), while feature sets without temperature (X4 and X9) lead to degraded performance across all metrics. This indicates a strong dependency of the model on temperature and derived features.

However, in the reduced scenario excluding NORA BLE and WiFi platforms, the overall performance of Random Forest models is generally comparable or slightly inferior to that of linear regression, with higher variability across repeated runs. This suggests that, despite their flexibility, Random Forest models do not consistently outperform linear models under limited data conditions.

The strong reliance on temperature-related features, combined with the small dataset size, may indicate partial overfitting. As a result, while Random Forest can capture more complex relationships, linear regression models with engineered features provide a more robust and reliable solution for the considered edge-oriented platform selection system.

## 11. Platform Selection Use Case

The regression-based framework supports decision-making between WiFi, BLE, and LoRa. This comparative capability is particularly valuable in adaptive systems, where transmission requirements may change over time and a static technology choice may lead to suboptimal energy performance.

Beyond platform and technology selection, the model can also be used to evaluate the impact of transmission parameter choices. Adjustments in throughput and transmission frequency directly influence the predicted energy cost, allowing exploration of trade-offs between data freshness, latency, and energy consumption.

The temperature emerges as a non-negligible factor influencing energy consumption, particularly through interaction with transmission activity. Although the temperature range observed during the experiments was limited, the inclusion of temperature-dependent interaction terms consistently improved the robustness of the decision in scenarios where the energy differences between the platforms were subtle. This suggests that environmental factors should not be ignored in energy modeling, especially in deployments exposed to varying thermal conditions.

Finally, the analysis underscores the limitations of simplified energy models based solely on average current values or static assumptions. Such models do not capture the combined effects of link variability, traffic dynamics, and environmental conditions.

## 12. Limitations and Discussion

The results presented in this study should be interpreted in light of several limitations related to the experimental design, data collection process, and modeling assumptions.

First, the measurement campaign was conducted over a limited time span of two days. As a consequence, the dataset does not capture long-term temporal variations in environmental conditions, network load, or interference patterns that may occur in real-world deployments. Although the repeated training and evaluation procedure demonstrates that the proposed models are stable with respect to random data partitioning, the results do not explicitly account for seasonal effects.

Second, the proposed regression models are empirical in nature and are not intended to represent a physical model of wireless energy consumption. The relationship between input features and current consumption is trained directly from measurement data, and the selected features serve as proxies for the underlying physical and protocol-level phenomena. For ESP32-based platforms, current consumption shows stronger dependence on transmission parameters and temperature, especially for Wi-Fi, where protocol dynamics introduce non-linear effects. This explains the benefits of richer feature sets and more expressive models. In contrast, NORA-based platforms operating with BLE and Wi-Fi exhibit relatively stable energy consumption, which reduces sensitivity to feature selection and allows linear models to achieve comparable performance. A different behavior is observed for LoRa communication, where energy consumption depends strongly on transmission parameters, making non-linear features more important. Overall, model effectiveness is closely linked to the variability of the energy process: stable profiles favor simpler models, while more dynamic conditions benefit from richer feature representations.

An additional limitation of the proposed approach is the use of RMS current measurements instead of instantaneous current waveform analysis. Although the adopted method captures steady-state energy consumption at the system level, it does not fully represent short-term transient effects such as retransmissions or contention mechanisms. This limitation may be particularly relevant for Wi-Fi communication, where dynamic MAC- and PHY-layer behavior can introduce rapid fluctuations in power consumption. Consequently, the lack of fine-grained transient information may reduce prediction accuracy under highly dynamic communication conditions. Nevertheless, the RMS-based representation significantly reduces measurement complexity and computational overhead, making the proposed framework more suitable for lightweight deployment on resource-constrained IoT edge devices.

An important limitation of the proposed study is the relatively small number of aggregated measurement configurations, which may reduce the generalization capability of the developed regression models under previously unseen operating conditions or communication scenarios. Nevertheless, the adopted lightweight linear regression approach was intentionally selected due to its low model complexity and reduced data requirements compared to more advanced machine-learning techniques. In addition, the dataset was obtained from a measurement campaign covering multiple wireless technologies and varying communication conditions, which improves the practical relevance of the collected data. The considered feature space also remains relatively low-dimensional, with a maximum of 11 input features, thereby reducing the risk of excessive model complexity and overfitting and making the selected regression models well suited for the considered task.

Finally, the experimental evaluation was limited to a specific set of hardware platforms and radio configurations. Although the selected platforms are representative of commonly used IoT devices, trained models cannot be directly generalized to other hardware without retraining. Differences in radio front-ends, antenna designs, power management architectures, and firmware implementations can lead to substantially different energy consumption characteristics.

Nevertheless, the proposed methodology—including feature selection, model formulation, and decision-oriented evaluation—remains applicable to other platforms and can be reused as a blueprint to construct similar models in different hardware contexts.

## 13. Conclusions

This paper presented a framework for the selection of energy-sensitive wireless communication platforms in IoT systems, based on empirical modeling of current consumption under varying operating conditions. The proposed approach combines systematic measurement, structured feature engineering, and lightweight regression models to predict current consumption and support comparative evaluation of candidate platforms. By focusing on aggregated metrics and statistically meaningful descriptors of link quality and load, the framework enables practical energy estimation without requiring low-level current waveform analysis or detailed protocol modeling.

A key contribution of this work is the construction of an empirical dataset that covers a broad range of operational configurations, including variations in distance, throughput, and transmission frequency, across multiple hardware platforms and communication technologies. The use of platform-specific regression models, combined with a structured feature ablation analysis, demonstrated that carefully selected interaction features can significantly improve decision quality in scenarios where energy differences between platforms are condition-dependent.

It should be noted that, despite the potential of the system described in the article, its main limitation was the limited scope of the experimental conditions. Therefore, future work will focus on expanding the dataset to include additional communication scenarios, environmental conditions, and mobility-related measurements in order to further improve the robustness and generalizability of the proposed framework.

## Figures and Tables

**Table 1:** Comparison of selected related works with respect to methodology, application focus, limitations, and differences from the proposed approach.

Ref.	Year	Methodology	Main Focus	Main Limitation	Difference from This Work
[4,5,6,7]	2018, 2018, 2017, 2021	Analytical energy modeling	Wireless communication energy consumption modeling	Requires detailed protocol-level analysis and focuses only on one technology	Uses lightweight statistical models and supports different wireless technologies
[8]	2018	Adaptive network configuration	Dense LoRa deployments	Network-oriented optimization	Proposed work focuses on communication platform selection
[9]	2019	Deep learning	Energy and reliability prediction	High computational requirements	Low computational requirements designed for edge implementation
[10,11,12]	2025, 2023, 2026	Machine learning models	Energy-aware	Focus on inference optimization, task allocation or routing	Proposed work focuses on communication platform selection
[13]	2016	Radio access technology selection	Heterogeneous networks	Focus on throughput and QoS metrics	Focus on energy-aware IoT operation
[14]	2005	Mathematical optimization	WLAN/UMTS network selection	Centralized and infrastructure-oriented	Proposed framework designed for edge implementation

**Table 2:** Experimental parameters registered during measurements.

Parameter	Description	Range/Values	Unit
RSSI (WiFi)	Observed received signal strength for WiFi	170 to 230	dBm
RSSI (BLE)	Observed received signal strength for BLE	140 to 200	dBm
RSSI (LoRa)	Observed received signal strength for LoRa	170 to 230	dBm
Throughput	Transmission throughput.	500, 1000, 2000, 4000	B/s
Frequency	Transmission frequency	0.1, 1, 5, 10	Hz
Temperature	Ambient temperature	−1 to 9	°C
Distance	Distance between IoT node and gate	5, 10, 25, 100, 225	m
RMS current	Current consumption of IoT node	25 to 122	mA

**Table 3:** Base input variables used in the regression models.

Variable	Unit	Description
RSSI_median_	dBm	Median received signal. strength indicator value.
RSSI_std_	dBm	Standard deviation of RSSI values.
Throughput	B/s	Transmission throughput.
Frequency	Hz	Transmission frequency.
Temperature	°C	Ambient temperature.

**Table 4:** Derived and interaction features used in the regression models.

Feature Name	Definition	Interpretation
RSSI^2^	${\left(RSSI_{median}\right)}^{2}$	Approximates the non-linear increase in energy consumption under degrading radio propagation conditions.
log(Throughput)	ln(Throughput+1)	Compresses the dynamic range of throughput values and models diminishing marginal energy cost with increasing data rate.
log(Frequency)	ln(Frequency+1)	Reduces the dominance of high transmission frequencies and stabilizes feature scaling.
Frequency/Throughput	$\frac{Frequency}{Throughput+1\;\times \;10^{-6}}$	Represents relative transmission load and duty-cycle intensity of the system.
RSSI × Throughput	$RSSI_{median}\cdot Throughput$	Captures the interaction between channel quality and transmitted data volume.
Temperature × Frequency	Temperature·Frequency	Models the interaction between environmental conditions and operational intensity.

## Data Availability

The raw data supporting the conclusions of this article will be made available by the authors on request.

## Abbreviations

The following abbreviations are used in this manuscript:

BLE	Bluetooth Low Energy
LoRa	Long Range
IoT	Internet of Things
RMSE	Root Mean Square Error
